# A Rare Metastatic Squamous Cell Carcinoma of the Lacrimal Sac Originating from Nasopharyngeal Carcinoma: A Case Report

**DOI:** 10.3390/reports9010041

**Published:** 2026-01-28

**Authors:** Vasileios Papanikos, Spyridon Lygeros, Athanasios Vlachodimitropoulos, Michail Athanasopoulos, Stylianos Mastronikolis, Nicholas S. Mastronikolis

**Affiliations:** 1 Department of Otolaryngology–Head and Neck Surgery, General University Hospital of Patras, 26504 Patras, Greece; papanikos.vas@gmail.com (V.P.); slygeros@upatras.gr (S.L.); miathanasopoulos@gmail.com (M.A.);; 2 Department of Ophthalmology, General University Hospital of Patras, 26504 Patras, Greece

**Keywords:** dacryocystitis, lacrimal sac metastasis, nasopharyngeal carcinoma, squamous cell carcinoma

## Abstract

**Background and Clinical Significance**: Metastatic carcinoma of the lacrimal sac originating from primary nasopharyngeal carcinoma (NPC) is a rare entity, usually presenting with chronic, unilateral epiphora. **Case Presentation**: A 55-year-old male patient presented with symptoms of chronic persistent dacryocystitis of the left eye for a year. His history revealed a non-keratinizing NPC diagnosed 5 years earlier, which was treated with combined radiotherapy (RT) and chemotherapy (CMT). Following CT and MRI scans, a mass was identified at the left lacrimal sac suggestive of a neoplasm in that region. The patient underwent endoscopic dacryocystorhinostomy (DCR), with tissue samples taken for biopsy. The histopathological diagnosis revealed a metastatic carcinoma of the lacrimal sac originating from the nasopharynx. The postoperative course was uneventful. However, a follow-up positron emission tomography-computed tomography (PET-CT) scan showed a hypermetabolic lesion in the left orbital cavity, infiltration of the lacrimal sac, hypermetabolic lateral cervical lymph nodes (IIA-IIB), and a hypermetabolic parotid lymph node. The patient is currently receiving combined CMT and immunotherapy (IMT) and is scheduled to receive RT thereafter. **Conclusions**: The non-specific symptomatology of the disease might be a reason for delayed diagnosis. Early recognition requires a high index of suspicion, while therapy mainly focuses on RT, CMT, IMT, and rarely on surgical approaches. A multidisciplinary approach and coordination are indispensable for the best possible treatment outcome.

## 1. Introduction and Clinical Significance

Nasopharyngeal carcinoma (NPC) is an epithelial malignancy of the head and neck that originates in the nasopharynx and is strongly associated with the Epstein–Barr virus (EBV) infection with a distinct geographical distribution, particularly prevalent in Southeast Asia [1,2,3]. Beyond its well-established association with EBV, NPC is increasingly recognized as a complex disease arising from dynamic interactions among viral factors, host genetics, environmental exposures, and the tumor microenvironment. Recent conceptual frameworks have proposed viewing NPC as an ecological disease, in which tumor progression, metastasis, and treatment resistance reflect adaptive and evolutionary processes within a heterogeneous biological ecosystem. This perspective provides a useful explanation for tumor dormancy, delayed recurrence, and atypical metastatic patterns, underscoring the need for long-term surveillance and heightened clinical awareness in NPC survivors [4]. NPC is characterized by an aggressive nature with local invasion and distant metastasis [3,5]. NPC poses a significant clinical challenge. While distant metastasis can occur in various sites, involvement of the lacrimal sac is extremely rare. Primary lacrimal sac tumors themselves are relatively uncommon, occurring in less than 1% of all lacrimal system neoplasms [6], with squamous cell carcinoma (SCC) being the most frequent subtype. The clinical presentation of lacrimal sac SCC often mimics that of chronic dacryocystitis, leading to possible delays in diagnosis and treatment [6,7].

This article describes an unusual case of metastatic SCC of the lacrimal sac from a primary nasopharyngeal carcinoma, highlighting the diagnostic difficulties, the need for a high index of clinical suspicion, and the value of multidisciplinary management of this rare clinical entity.

## 2. Case Presentation

A 55-year-old male patient presented to our ENT outpatient department with symptoms of unilateral dacryocystitis for the past year. Specifically, he reported epiphora of the left eye, corresponding eye redness, swelling, and difficulty in opening the eye in the morning upon waking.

The patient’s history revealed hyperlipidemia and tachyarrhythmia treated with atorvastatin and propafenone, respectively. In addition, he had a history of non-keratinizing SCC of the nasopharynx diagnosed 5 years earlier, for which he underwent 20 cycles of chemotherapy (CMT) and 35 sessions of radiotherapy (RT). Moreover, he had undergone appendectomy and lower extremity fracture reduction in the past. He was a non-smoker and drank alcohol socially.

**Figure 1:**
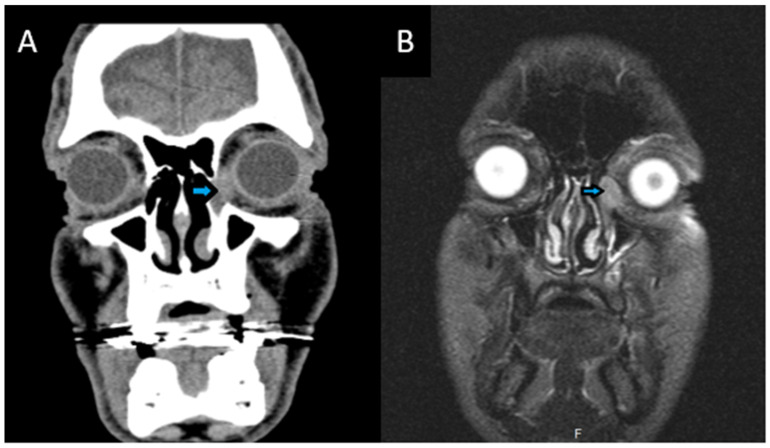
(**A**) Coronal CT scan of the craniofacial region demonstrating mild swelling (arrow) in the left lacrimal sac region. (**B**) Coronal T2-weighted MRI of the orbits showing a well-defined mass with high signal (arrow) in the left lacrimal sac.

All laboratory test results were within normal limits. A computed tomography scan (CT) of the craniofacial region was performed and revealed a mild swelling in the area of the left lacrimal sac and mild mucosal thickening at the left maxillary sinus (Figure 1). Subsequent magnetic resonance imaging (MRI) showed an ovoid-shaped formation, measuring approximately 14 × 13 × 10 mm, in the left orbital cavity and in an extraconal location, at the site of the lacrimal sac (Figure 1). The mass exhibited significant and homogeneous vascularization with well-defined margins. The extraocular muscles were unremarkable, and the optic nerve showed no abnormalities. The findings were consistent with a neoplasm at the level of the lacrimal sac.

**Figure 2:**
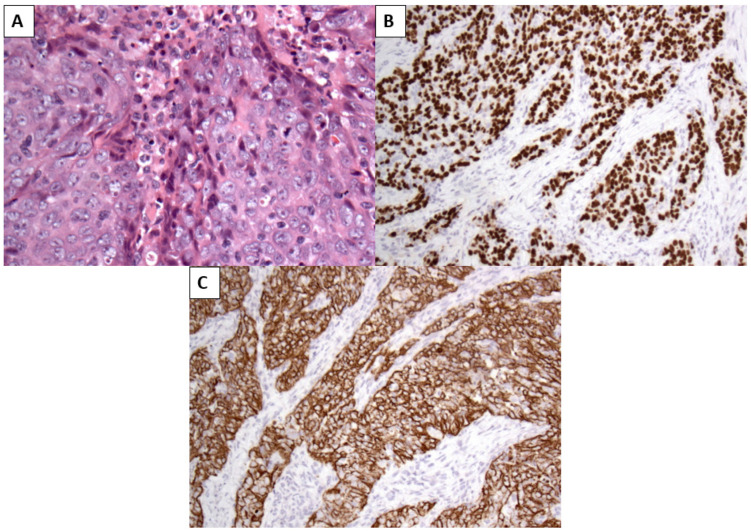
(**A**) Hematoxylin- and eosin-stained section showing a non-keratinizing squamous cell carcinoma composed of sheets and nests of malignant epithelial cells with indistinct cell borders, vesicular nuclei, and prominent nucleoli, embedded in a lymphocyte-rich stromal background, consistent with the morphology of nasopharyngeal carcinoma (original magnification ×200). (**B**) Immunohistochemical staining demonstrating strong and diffuse positivity for p63 (**C**) and Cytokeratins 5/6 in the tumor cells, confirming squamous differentiation (original magnification ×200).

The patient underwent endoscopic dacryocystorhinostomy (DCR) with excision of the mass, which was submitted for histopathological examination, along with probing of the lacrimal puncta. Histopathological examination revealed a non-keratinizing SCC infiltrating the lacrimal sac, characterized by sheets of malignant epithelial cells with indistinct cell borders and a prominent lymphocyte-rich stroma, closely resembling the histological features of the patient’s previously diagnosed NPC. Immunohistochemical analysis demonstrated strong nuclear positivity for p40, p63, and cytokeratins 5 and 6, confirming squamous differentiation (Figure 2). Despite the lack of detectable local recurrence at the primary nasopharyngeal site, the morphological similarity to the original tumor and the clinicopathological context strongly supported a diagnosis of metastatic NPC. Epstein–Barr Virus-encoded RNA (EBER) in situ hybridization was not performed, representing a limitation of the present case.

**Figure 3:**
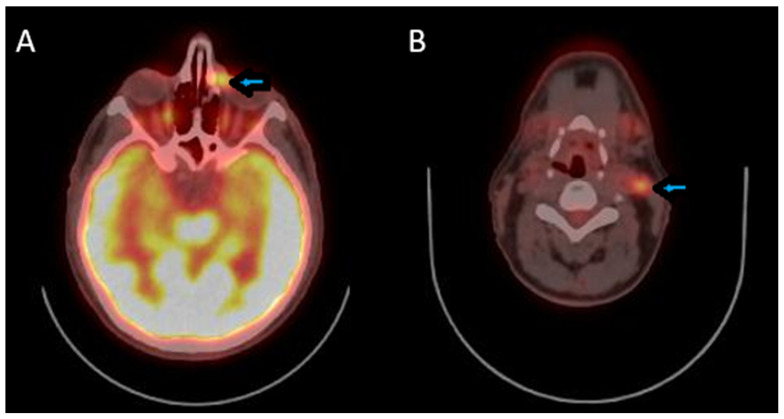
(**A**) PET-CT scan indicating a hypermetabolic lesion (arrow) in the extraconal compartment of the left orbital cavity, consistent with metastatic disease. (**B**) PET-CT showing hypermetabolic lymph node (arrow) in the left lateral cervical region, levels IIA–IIB, suggestive of metastatic involvement.

During follow-up, the patient had an uneventful postoperative course, without complications or residual symptoms. As part of the postoperative evaluation, a Positron Emission Tomography–Computed Tomography (PET-CT) scan was performed, which revealed a hypermetabolic lesion in the extraconal compartment of the left orbital cavity with infiltration of the lacrimal sac, hypermetabolic lymph nodes in the left lateral cervical region (levels IIA-IIB), and the presence of a hypermetabolic left parotid lymph node (Figure 3). The patient is undergoing combined CMT (cisplatin) and immunotherapy (IMT) (nivolumab) and is scheduled to receive radiotherapy thereafter.

**Figure 4:**
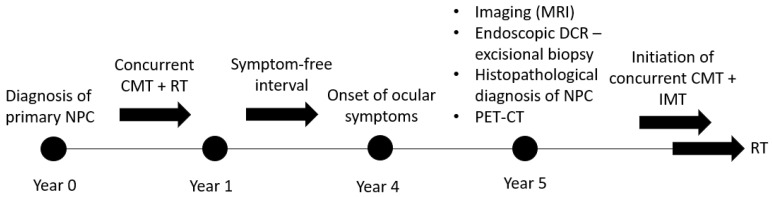
Key stages in the patient’s clinical evolution, including the initial diagnosis and treatment of nasopharyngeal carcinoma, the delayed onset of unilateral ocular symptoms, radiological identification of a lacrimal sac lesion, histopathological confirmation of metastatic disease, and subsequent systemic staging and treatment.

To facilitate a clear understanding of the chronological progression of the disease and its management, a timeline summarizing the key clinical events of the present case is provided in Figure 4.

## 3. Discussion

Nasopharyngeal carcinoma, an epithelial malignancy of the nasopharynx strongly associated with EBV, has a strong geographic association, particularly in Southeast Asia, Alaska, and North Africa [1,3]. EBV infects more than 90% of the global population, mainly during childhood or adolescence [2], and later enters a latent state in B lymphocytes and epithelial cells, with its reactivation being associated with several diseases, including malignancies [8]. NPC is classified into various histological subtypes, including keratinizing or non-keratinizing SCC, with the latter being associated with EBV in nearly all cases [1,2]. The non-specific symptoms of NPC, including headache, epistaxis, and facial pain, make early detection of NPC a significant challenge, with 70% of NPC patients being diagnosed at a locally advanced stage [9]. NPC is recognized for its propensity to metastasize to regional lymph nodes and distant organs (nearly 30% of cases), including bones (most commonly), lungs, and liver, mainly within the first two years after treatment [3,5,10,11,12]. However, metastasis to the lacrimal sac is exceedingly rare, with an incidence rate even lower than that of primary lacrimal sac SCC (<1%) [6]. SCC is the most frequent histological subtype, keratinizing or non-keratinizing, which mainly occurs in middle-aged patients, typically over 50 years [6,13,14,15]. Various risk factors have been associated, such as EBV, human papillomavirus, infections, and chronic inflammation [13,16]. Its most common symptoms include unilateral epiphora (70%) and the presence of a palpable mass in the area of the medial canthus, while the less common (4–10%) pain and bloody epiphora often indicate advanced disease [7,13]. All of the above symptoms can mimic benign conditions, which may lead to a delayed diagnosis. On average, the time from symptom onset to confirmed diagnosis is approximately three years [6,7]. In contrast to the literature, in our case, ocular symptoms developed approximately four years after completion of treatment for the primary tumor, with the diagnosis of lacrimal sac metastasis established five years after the initial NPC diagnosis. This prolonged interval highlights the importance of long-term surveillance of NPC patients, even after apparently successful treatment of the primary site.

Additionally, persistent dacryocystitis, especially unilateral, should raise suspicion of malignancy and must be thoroughly investigated. The diagnostic challenge encountered in this case highlights the necessity of having a high index of suspicion for metastatic disease in patients with a history of NPC presenting with ocular symptoms. The initial presentation was in the form of chronic dacryocystitis and led to delayed diagnosis. This underscores the need for thorough evaluation, such as CT and MRI, in patients presenting with chronic or atypical ocular symptoms, especially those with a past history of head and neck cancer [7]. Our case also demonstrates the use of PET-CT in identifying secondary sites of metastatic disease and guiding treatment planning, as it revealed unsuspected lymph node disease.

Furthermore, the isolated involvement of the lacrimal sac in the absence of concurrent metastasis to adjacent orbital or nasal tissues, as seen in our case, is a rare pattern of spread. The disease has often spread to other anatomical locations as well, such as the upper jugular lymph nodes, lungs, and liver [6,17]. The close anatomical proximity of the nasopharynx to the lacrimal drainage system likely accounts for this metastatic pathway; the mechanisms, however, are not yet clearly outlined.

Regarding prognosis, while the overall prognosis for metastatic NPC is generally good [13], the patient’s specific prognosis is influenced by several factors, including the five-year interval between the primary NPC diagnosis and the lacrimal sac metastasis, as well as the presence of other metastatic lesions. The reported recurrence rate for lacrimal sac neoplasms ranges from 20 to 50%, with mortality between 10 and 40% [13]. However, in the context of metastatic NPC, these figures may not be directly applicable, and the patient’s long-term outcome remains uncertain. Long-term follow-up is therefore crucial to monitor for disease progression or recurrence.

The treatment of metastatic lacrimal sac SCC from NPC is complex and can be multidisciplinary in approach [15,18,19]. In our patient, there was a surgical excision of the mass in the lacrimal sac, which was deemed appropriate for diagnostic biopsy and according to the initial assessment of localized disease. Following surgery, however, PET-CT scan revealed additional metastatic diseases including hypermetabolic lymph nodes. Accordingly, the patient is currently under combined CMT and IMT based on standard guidelines for NPC and considering the patient’s overall performance status [20]. The patient is also scheduled to undergo RT upon completion of the initial regimen. The use of combined modalities of treatment was considered necessary to manage both the local recurrence and systemic spread of the disease.

This case report has certain limitations that should be acknowledged. Epstein–Barr virus-encoded RNA in situ hybridization and circulating EBV DNA quantification were not available at our healthcare facility at the time of diagnosis and therefore were not performed. As a result, the diagnosis relied on characteristic histomorphological features, immunohistochemical findings, clinicopathological correlation, and systemic staging with PET-CT.

## 4. Conclusions

This case highlights the diagnostic challenge posed by lacrimal sac involvement in survivors of nasopharyngeal carcinoma, as its non-specific presentation may closely mimic benign conditions such as chronic dacryocystitis. The prolonged interval between primary treatment and metastatic presentation underscores the necessity of long-term surveillance and a high index of clinical suspicion in NPC patients presenting with unilateral ocular symptoms. Early recognition through appropriate imaging and histopathological evaluation, together with multidisciplinary management, is essential for timely diagnosis and optimal treatment.

## Data Availability

The data that support the findings of this study are available on request from the corresponding author. The data are not publicly available due to privacy or ethical restrictions.

## Abbreviations

The following abbreviations are used in this manuscript:

NPC	Nasopharyngeal Carcinoma
EBV	Epstein–Barr Virus
SCC	Squamous Cell Carcinoma
CMT	Chemotherapy
IMT	Immunotherapy
RT	Radiotherapy
CT	Computed Tomography
MRI	Magnetic Resonance Imaging
DCR	Dacryocystorhinostomy
EBER	Epstein–Barr Virus-encoded RNA
PET-CT	Positron Emission Tomography–Computed Tomography
