# Correction: Papanikos et al. A Rare Metastatic Squamous Cell Carcinoma of the Lacrimal Sac Originating from Nasopharyngeal Carcinoma: A Case Report. *Reports* 2026, *9*, 41

**DOI:** 10.3390/reports9010094

**Published:** 2026-03-23

**Authors:** Vasileios Papanikos, Spyridon Lygeros, Athanasios Vlachodimitropoulos, Michail Athanasopoulos, Stylianos Mastronikolis, Nicholas S. Mastronikolis

**Affiliations:** 1 Department of Otolaryngology–Head and Neck Surgery, General University Hospital of Patras, 26504 Patras, Greece; papanikos.vas@gmail.com (V.P.); slygeros@upatras.gr (S.L.); miathanasopoulos@gmail.com (M.A.); nmastr@otenet.gr (N.S.M.); 2 Department of Ophthalmology, General University Hospital of Patras, 26504 Patras, Greece; s.mastronikolis@nhs.net

In the original publication [1], an error was identified in Reference [4].

The correct reference is provided below.

4. Luo, W. Nasopharyngeal carcinoma ecology theory: Cancer as a multidimensional spatiotemporal “unity of ecology and evolution” pathological ecosystem. *Theranostics* **2023**, *13*, 1607–1631. https://doi.org/10.7150/thno.82690.

The authors state that the scientific conclusions are unaffected.

This correction was approved by the Academic Editor.

The original publication has also been updated.
